# Molecular Screening for Cyanobacteria and Their Cyanotoxin Potential in Diverse Habitats

**DOI:** 10.3390/toxins16080333

**Published:** 2024-07-27

**Authors:** Maša Jablonska, Tina Eleršek, Polona Kogovšek, Sara Skok, Andreea Oarga-Mulec, Janez Mulec

**Affiliations:** 1 Department of Genetic Toxicology and Cancer Biology, National Institute of Biology, 1000 Ljubljana, Slovenia; tina.elersek@nib.si; 2 Biotechnical Faculty, University of Ljubljana, 1000 Ljubljana, Slovenia; 3 Department of Biotechnology and Systems Biology, National Institute of Biology, 1000 Ljubljana, Slovenia; polona.kogovsek@nib.si; 4 Karst Research Institute, Research Centre of the Slovenian Academy of Sciences and Arts, 6230 Postojna, Slovenia; sara.skok@zrc-sazu.si; 5 Materials Research Laboratory, University of Nova Gorica, 5000 Nova Gorica, Slovenia; andreea.oarga@ung.si; 6 UNESCO Chair on Karst Education, University of Nova Gorica, 5271 Vipava, Slovenia

**Keywords:** extreme environments, cylindrospermopsin, sulphidic springs, caves, qPCR, PC-IGS

## Abstract

Cyanobacteria are adaptable and dominant organisms that exist in many harsh and extreme environments due to their great ecological tolerance. They produce various secondary metabolites, including cyanotoxins. While cyanobacteria are well studied in surface waters and some aerial habitats, numerous other habitats and niches remain underexplored. We collected 61 samples of: (i) biofilms from springs, (ii) aerial microbial mats from buildings and subaerial mats from caves, and (iii) water from borehole wells, caves, alkaline, saline, sulphidic, thermal, and iron springs, rivers, seas, and melted cave ice from five countries (Croatia, Georgia, Italy, Serbia, and Slovenia). We used (q)PCR to detect cyanobacteria (phycocyanin intergenic spacer—PC-IGS and cyanobacteria-specific 16S rRNA gene) and cyanotoxin genes (microcystins—*mcyE*, saxitoxins—*sxtA*, cylindrospermopsins—*cyrJ*), as well as amplicon sequencing and morphological observations for taxonomic identification. Cyanobacteria were detected in samples from caves, a saline spring, and an alkaline spring. While *mcyE* or *sxtA* genes were not observed in any sample, *cyrJ* results showed the presence of a potential cylindrospermopsin producer in a biofilm from a sulphidic spring in Slovenia. This study contributes to our understanding of cyanobacteria occurrence in diverse habitats, including rare and extreme ones, and provides relevant methodological considerations for future research in such environments.

## 1. Introduction

Cyanobacteria, the earliest photosynthetic life forms and often referred to as “ecosystem engineers” [1], stand out as primary colonizers. They are dominant in diverse environments, including harsh and extreme conditions due to their long-term evolution, great ecological tolerance, morphological plasticity, and adaptability. Harsh and extreme environments, characterized by extreme temperature, pressure, salinity, ionizing radiation, pH, and high metal/salt levels, are challenging for organisms [2,3,4]. Cyanobacteria synthetize diverse biomolecules that help them to survive under such conditions [5]. They have evolved robust mechanisms to protect the photosynthetic machinery from desiccation [6], specialized pigments to capture a broad spectrum of light, and robust cell walls to withstand environmental fluctuations. Their ability to fix nitrogen provides essential nutrients in nitrogen-poor areas [7], while some species produce protective mucilage that protects them from desiccation [8]. They can also tolerate extreme pH levels and high salinity. Adaptability and remarkable resistance to desiccation and radiation also ensure survival in harsh environments [9]. Recent advances in metagenomics and experimental research have brought to light significant metabolic adaptations in response to changing conditions such as desiccation/rehydration cycles. These adaptations include production of extracellular polysaccharides, enhanced DNA repair, formation of protein complexes to maintain integrity, adaptation to low water activity through compatible solute synthesis and regulation of ion channels, and protection against oxidative stress [10,11,12].

As a result, cyanobacteria have been found in polar regions, hot deserts, alkaline lakes, acidic environments [13], hypersaline environments, hot springs [14], caves, ice, snow [15], and ambient springs, i.e., springs with a temperature approaching the mean annual air temperature in the catchment area [16]. It has been shown that cyanobacteria can tolerate an extreme stratospheric environment similar to Mars [17] and that certain species such as *Chroococcidiopsis* are a promising model for synthetic biology in space [9]. Whether a species can be a successful colonizer in the long term depends not only on its physiological fitness, but also on the presence of and interaction with other species and suitable environmental parameters [18]. For some species, their frequency and abundance are largely influenced by human activities [19].

Cyanobacteria produce various secondary metabolites, including different groups of cyanotoxins that cause toxic effects in vertebrates [20] and bioaccumulate in biomass and other material through absorption processes [21]. Due to the toxicity of these compounds and their occurrence in aquatic environments, the World Health Organization has established guidelines for drinking and recreational water for the most commonly occurring cyanotoxins worldwide [22]. The presence of cyanotoxins has been confirmed in many water habitats [23], but a complete list is still missing. The most common cyanotoxins in water environments are hepatotoxic microcystins and nodularins, hepato- and cytotoxic cylidrospermopsins, and neurotoxic anatoxins and saxitoxins [24,25]. Microcystins are toxic to humans, molluscs, crustaceans, fish, amphibians, mammals, and birds, and have adverse effects in the form of hepatotoxicity, reproductive toxicity, cardiotoxicity, neurotoxicity, immunotoxicity, and endocrine disruption [26]. Cylindrospermopsins can damage the liver, kidneys, and other organs, and may also have immunotoxic effects. Saxitoxins and anatoxins target the nervous system and lead to muscle paralysis and respiratory failure [20]. In addition, some of these toxins can be genotoxic [27]. While toxin-producing cyanobacteria are well studied in surface waters, much less is known about their occurrence in extreme and rare habitats, although they could also affect the associated biota and ecosystem services there. For example, cyanotoxins have been detected in the poorly studied lampenflora community (microbial mat that forms around lamps in show caves) in Mammoth Caves, USA [28] and in public hot springs used for bathing and recreation [29]. Cyanobacteria in hot springs have been associated with flamingo poisoning [30]. Recent advances in molecular methods allow us to study such environments using microbial community DNA. This can provide information on community composition (e.g., [31]), functional traits (e.g., [19]), or the ability to produce certain secondary metabolites, such as cyanotoxins (e.g., [32]).

Cyanobacteria, a phylum of bacteria, have recently received more scientific attention to better understand their ecology and role in various habitats such as freshwater, marine ecosystems, soils, rocks, and extreme environments such as hot springs and polar regions. They can be a constitutive or even dominant component of biofilms, microbial mats, and aquatic habitats. A microbial mat is a multi-layered complex structure of microorganisms, in which different layers harbour different microbial populations that carry out different metabolic processes. A biofilm is a structured community of microorganisms embedded in a self-produced extracellular polymeric substance that protects the community from environmental stresses. Aquatic ecosystems can range from common habitats, such as rivers, lakes, or the sea, to rare environments, such as cave water and cave ice, or springs with extreme environmental conditions (e.g., alkaline, saline, or thermal springs). Cyanotoxins have been detected in all these sample types—microbial mats, biofilms, and water—in various rare habitats, but studies are sparse and geographically limited [14].

In this study, we analysed randomly selected and accessible sites in different geographical locations in Europe for the presence of cyanobacteria and cyanotoxin genes. These include various water and microbial mat samples from common habitats, habitats with rare distribution, and habitats known for extreme environmental parameters. We analysed samples of biofilms from springs, aerial microbial mats from buildings and subaerial mats from caves, and water from borehole wells, caves, alkaline, saline, sulphidic, thermal, and iron springs, rivers, the sea, and melted cave ice. The reason for selecting the sites was an initial screening to determine the potential distribution of cyanobacteria and the cyanotoxin potential in different habitats that have not yet been investigated using molecular approach. Samples were screened for the presence of major cyanotoxin genes using end-point PCR, quantitative PCR (qPCR), and amplicon sequencing, which supported and expanded the existing knowledge of cyanobacteria occurrence. The results confirmed the presence of cyanobacteria in caves, a saline spring, and an alkaline spring. Molecular screening for cyanotoxins did not reveal the presence of microcystin and saxitoxin synthesis genes in any of the samples, but suggested a potential occurrence of cylindrospermopsin producers in biofilm from a sulphidic spring.

## 2. Results and Discussion

### 2.1. Molecular Detection of Cyanobacteria: Findings and Constraints

**Figure 1:**
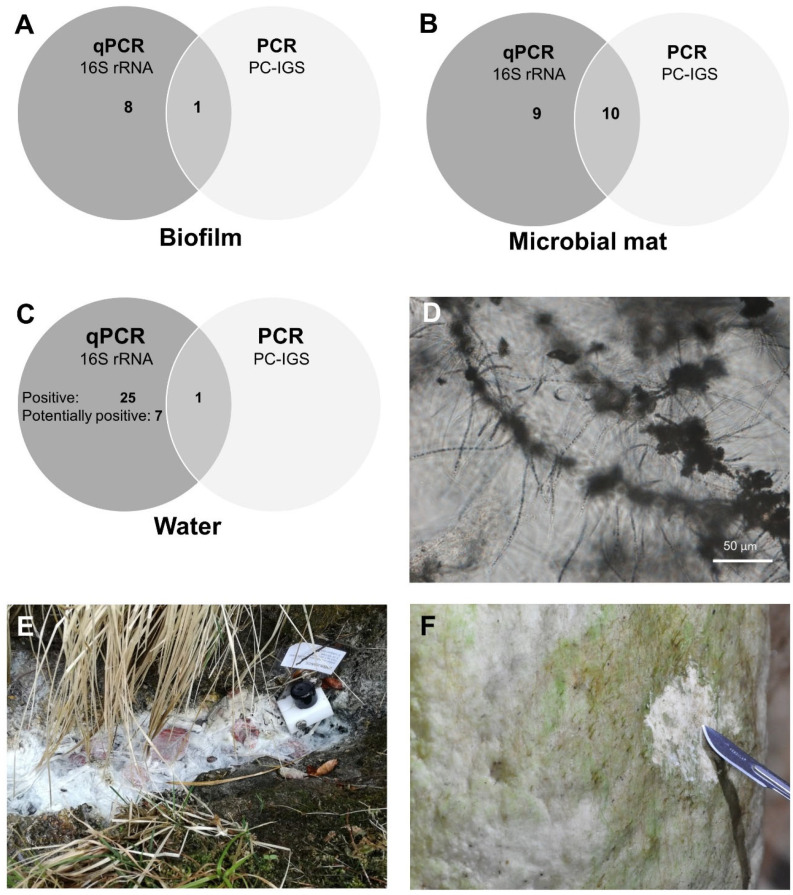
Comparison of molecular tests qPCR (16S rRNA in autotrophs) and PCR (PC-IGS—phycocyanin intergenic spacer) for detection of cyanobacteria for different sample types: biofilm (**A**), microbial mat (**B**), water (**C**). Number of positive samples determined with each method and with both methods is given. (**D**) a microphotograph of a sample from Žveplenica sulphidic spring, Slovenia, with abundant filaments of sulphur-oxidizing bacteria *Thiothrix*. (**E**) white biofilm of Žveplenica sulphidic spring and temperature datalogger at spring’s orifice. (**F**) lampenflora in Postojnska jama.

Molecular analysis indicated the presence of autotrophs in all habitat types studied (Figure 1A–C, Table 1). Molecular signals for cyanobacteria-specific 16S rRNA gene were detected in waters with a pH of 2.5 to 12.4, a temperature range of 10 to 83 °C, in sulphidic water (~8.5 mg/L H_2_S in Žveplenica sulphidic spring [33]), cave ice [34], the sea, terrestrial saline spring (2 mg/L NaCl, unpublished data), cave seepage water, and discharge from borehole wells (Table 1). The screened microbial mats were collected in environments exposed to light. Their macroscopic greenish appearance indicated the presence of phototrophs (Figure 1F), which was confirmed by the molecular tests. In the biofilm samples, the presence of phototrophs was not so obvious, as none of the samples showed a colour typical of photosynthetic pigments (Figure 1E). Nevertheless, the initial qPCR screening with a cyanobacteria-specific 16S rRNA assay indicated the presence of autotrophs in 56 of the 61 samples (92%). The remaining five samples (8%), all originating from water, showed low signal (only one or two out of three replicates tested were positive) and were considered potentially positive (Figure 1C, Table 1). Fifty samples produced results within the quantification range, and the calculated cell concentrations of autotrophs ranged from 15 to 6.6 × 10^5^ cells per μL DNA, while the remaining 11 samples (two biofilm and nine water samples) were positive but below quantification limit (Supplementary Table S1).

**Figure 2:**
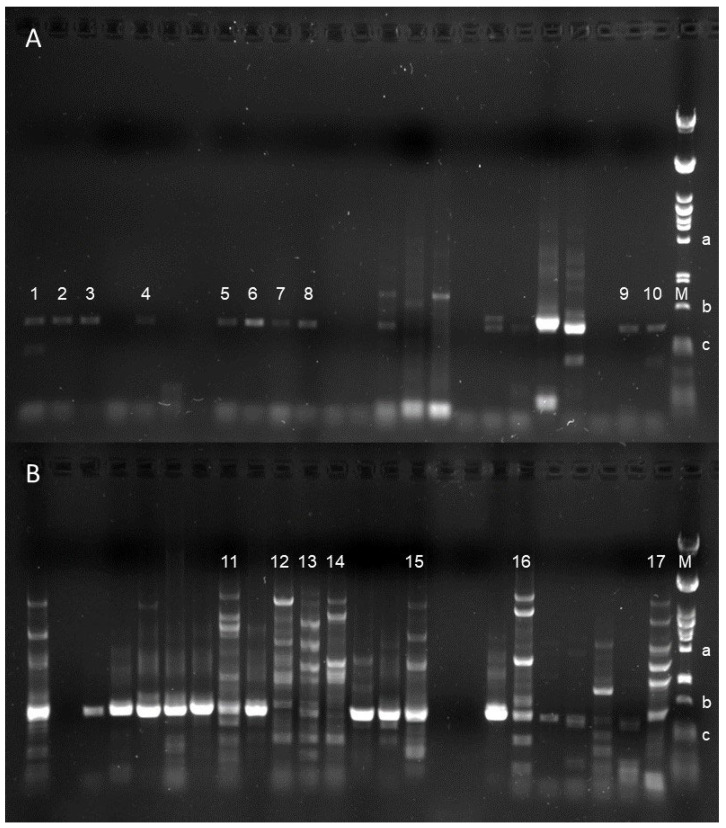
Examples of specific (**A**) and non-specific (**B**) PCR amplification products of the phycocyanin intergenic region visualized on 0.8% agarose gel containing 0.5 × Tris-Borate-EDTA buffer, stained with Midori Green Advance DNA Stain (NIPPON Genetics EUROPE, Düren, Germany) at 120 V for 60 min. Samples: 1—W-06, 2—W-11, 3—W-03, 4—W-05, 5—W-02, 6—W-04, 7—W-18, 8—W-19, 9—W-14, 10—W-12, 11—M-16, 12—M-07, 13—M-08, 14—M-09, 15—M-19, 16—M-04, 17—B-08 (see Table 1 for details). Size marker (M): pstI-digested λ DNA; a—1700 bp, b—805 bp, c—514 bp. Expected product size 500–740 bp.

To confirm the presence of cyanobacteria in the samples, phycocyanin intergenic spacer (PC-IGS) was amplified by PCR (Figure 2). Amplification products were observed in 34 of 60 samples (57%), but the results were inconclusive in many cases due to the presence of products of different lengths on the gel (Figure 2B), so amplification was repeated to confirm the results. The presence of multiple bands on the gel might be a consequence of non-specific amplification of other similar sequences of the bacterial genomes. In addition, a significant variation in fragment length is expected in this target region [35], and mixed populations of cyanobacteria are expected in environmental samples. However, twelve samples (20%) produced a distinct product of 500–800 bp in both repetitions and were therefore considered positive for PC-IGS with high confidence (Table 1), and sequencing of those amplicons was attempted. It should be noted that the remaining 22 samples showed a weak amplification product in the first repetition of the experiment, which was not reproducible in the second repetition. This could be explained by either a very low concentration of the target gene (stochastic effect—target region is randomly amplified or not) [36] or degradation of DNA due to multiple freeze/thaw cycles, and these samples were not considered positive.

The observed discrepancies between the results of the PC-IGS and 16S rRNA assays (samples positive for 16S rRNA gene only, Table 1) could be a consequence of cross-reactivity of the 16S rRNA qPCR assay with chloroplasts of plants or algae [37], resulting in the detection of eukaryotic algae in the samples. In addition, the high sensitivity of the qPCR and the design of qPCR assays to amplify short DNA fragments improve the probability of detecting target sequences compared to PCR. The PC-IGS PCR assay targets much longer fragments (over 500 bp) than the 16S rDNA qPCR assay (about 70 bp), meaning that qPCR could also amplify partially degraded DNA, and this could negatively affect PCR amplification [38].

The PC-IGS region has previously been used as a phylogenetic marker for cyanobacteria (e.g., [35,39,40]), as it is specific only to this taxa group, and carries sufficient genetic variation to distinguish between different taxa [41]. In previous studies, the PC-IGS-based phylogeny was mostly consistent with the 16S rRNA-based phylogeny [42,43,44], but showed better coverage and specificity compared to the 16S rRNA gene region [39], as well as better resolution between closely related strains [41,44,45]. In this study, the presence of the PC-IGS genetic region and thus the presence of cyanobacteria was confirmed by sequencing in seven of the twelve samples; five samples of microbial mat from caves (M-10, M-14, M-15, M-17, M-19 from Postojnska jama and Škocjanske jame) in Slovenia, one biofilm sample (B-01) from an alkaline spring in Serbia, and one water sample (W-01) from a saline spring in Croatia (Table 1). In four of these seven samples (B-01, M-10, M-14, M-15), taxa could not be reliably determined due to low read quality, indicating a mixed cyanobacterial community. The latter observation was already made during PCR amplification of this gene, where several PCR products were amplified (Figure 2B). Since Sanger sequencing approach is designed for single template reactions and not for mixed communities, amplicon cloning and sequencing of a number of clones would be required to determine the taxa from those mixed communities. Furthermore, there are significant gaps in the taxonomic completeness of reference databases [46], which is why some taxa may remain unidentified. While Salmaso et al. [46] highlighted the importance of this gap for the widely studied 16S rRNA gene, it is likely even more pronounced for PC-IGS with more than 25 times fewer records within the cyanobacterial phylum (NCBI Nucleotide, accessed 17 July 2023). Therefore, further studies on the phylogenetic composition of these samples, employing cloning of PCR products and sequencing of different phylogenetic markers, would be needed to clearly determine the composition of the cyanobacterial community.

In three of seven samples, a single cyanobacterial taxon was determined with PC-IGS sequencing. A taxon phylogenetically close to *Cyanothece* sp. was identified in an aerophytic microbial mat from the Škocjanske jame cave entrance (M-17; query cover 91–94%, identity 75–77%), and *Calothrix* sp. was identified in the community of a stromatolitic stalagmite from the same cave (M-19, query cover 100%, identity 91%). The presence of *Cyanothece* was previously confirmed by microscopic examination of field material from several sites in this cave [47], as well as *Calothrix* and other cyanobacterial taxa, e.g., *Aphanocapsa*, *Aphanothece*, *Chroococcus*, *Gloeocapsa*, *Homoeohtrix*, *Leptolyngbya*, *Lyngbya*, *Nostoc*, *Oscillatoria*, *Phormidium*, *Planktolyngbya*, *Pseudoanabaena*, *Schizohtrihy*, *Scytonema*, *Syhnecohcystis*, and *Trichodesmium* [48]. The presence of most of these taxa was also confirmed microscopically in other caves [49,50,51]. In a terrestrial saline spring, *Synechocystis* sp. was identified (W-01; query cover 100%, identity 91–93%) (Table 1). Some *Synechocystis* strains are known to be halotolerant, growing in saline or sea waters, or saline (brackish) swamps [52], and the marine strain *Synechocystis* sp. PCC 7338 was found to have several genes related to adaptation to high salinity and high osmotic pressure [53]. Other studies on saline springs have found *Calothrix pulvinata*, *Phormidium tergestinum*, *Tapinothrix violacea*, and *Rivularia* sp. aff. *bullata* to be the characteristic taxa in such environments [16], but we did not find these taxa in the studied terrestrial saline spring in Croatia.

Nevertheless, this study confirmed the presence of cyanobacteria in some extreme environments, e.g., in biofilms from alkaline and saline springs, for which there were no previous reports. However, additional and systematic sampling in different locations and sample types is needed to explore further the presence of cyanobacteria in such rare and diverse habitats.

### 2.2. Cylindrospermopsins: Potential Presence in Sulphidic Springs

The potential for cyanotoxin production (microcystin, saxitoxin, and cylindrospermopsin synthesis genes) was studied with different qPCR assays applied to all samples positive with either 16S rRNA gene, PC-IGS, or both. Microcystin and saxitoxin producers were not clearly detected in any sample (a weak qPCR signal for *Planktothrix*-specific *mcyE* gene was observed in the sample W-18 (Table 1), but this was not investigated further). However, the presence of cylindrospermopsin producers was indicated in four (7%) of the 61 samples (B-07, B-08, M-14, M-15; Table 1). The calculated cell concentrations of potential cylindrospermopsin producers were between 67 and 801 cells per μL DNA (Supplementary Table S2), but the qPCR results were inconclusive. To confirm the qPCR results, the *cyrJ* region was amplified by end-point PCR targeting a longer gene fragment, and a product of the expected length was detected and sequenced in two biofilm samples from Žveplenica (B-08) and Riharjev studenec (B-07) sulphidic springs. In Riharjev studenec, the 563 bp long fragment (100% overlap between F and R reads) showed 95% similarity to the sulphur-oxidizing bacteria *Thiothrix fructosivorans* ATCC 49748. The overlapping region corresponded to gene QTX09933.1 (NCBI Nucleotide) in *T. fructosivorans*, which has been annotated as a response regulator by automated computational analysis.

In Žveplenica, the taxa could not be reliably determined based on *cyrJ* sequence, but the results indicate the presence of organisms with genetic potential for cylindrospermopsin production. More specifically, the forward *cyrJ* read was full of overlapping bases, suggesting a mixed community, but the reverse *cyrJ* read was of good quality and matched cyanobacterial *cyrJ* genes in the NCBI GenBank database (*Oscillatoria* strains AWQC-PHO021 and PCC 6506, 95% identity). This may indicate that the forward primer coincidentally aligned with a DNA fragment from another organism, possibly *Thiothrix*. A previous molecular study on the biodiversity of Žveplenica sulphidic spring has shown that *Thiothrix* dominates the biofilm (Mulec and Summers Engel, 2019). To investigate this further, a microscopic analysis of the Žveplenica biofilm was performed. The microscopic examination revealed the presence of filaments belonging to the sulphur-oxidizing bacteria *Thiothrix* (Figure 1D); the only cyanobacterial taxon identified in the sample was *Tolypothrix*, but the identification was not straightforward, as the filaments were not clearly visible due to the abundant *Thiothrix* filaments. It is worth mentioning that some publications describe heterocysts in *Tolypothrix* [54], which were not observed morphologically in the Žveplenica sample.

Nevertheless, sulphidic springs are often colonized by thick aggregates of sulphur-oxidizing *Thiothrix* [55]. Microscopic observations of *Thiothrix* in Žveplenica and the sequencing results suggest that the positive signal of the *cyrJ* target region (forward read) could also originate from *Thiothrix*. To test whether this was due to non-specific primer alignment or homology, we blasted the QTX09933.1 gene of *Thiothrix fructosivorans* strain ATCC 49748 against the cyanobacteria/melaionabacteria group, and a *cyrJ* reference gene from *Cylindrospermopsis raciborskii* AWT205 (ABX60159.1) against *Thiothrix*/*Francisella* group in the NCBI Protein database (blastp), but no significant hits were found. While parts (13 bp) of primer sequences cynsulF and cylnamR (see 4.2. Molecular Analyses) matched 100% with various *Thiothrix* strains, alignment of the reference *cyrJ* gene from *Cylindrospermopsis raciborskii* AWT205 (EU140798.1) with the *Thiothrix* group from the NCBI Nucleotide database revealed only short matching fragments, so non-specific primer alignment and amplification of different genome fragments (due to low annealing temperature of the primers) is the more likely explanation. This explanation may apply not only to Žveplenica (B-08) but also to Riharjev studenec (B-07). The Žveplenica sulphidic spring has already been discovered as a biodiversity hotspot and a unique habitat with a rich and diverse microbial biofilm and copepods as the most abundant invertebrate species [33]. In this spring, the presence of cyanobacteria, e.g., *Oscillatoria*, has already been detected by molecular analyses [56], but their characteristics and role have not been explored. Cyanobacteria in microbial mats of sulphidic springs can simultaneously perform oxygenic and anoxygenic photosynthesis [57]. While the evolutionary history of metabolically versatile cyanobacteria remains unknown, data suggest the possibility of coevolution of sulphate reduction and anoxygenic cyanobacterial photosynthesis in microbial mat systems where the local sulphur cycle is driven by a dense biofilm population [58]. The existence of cyanotoxin-producing strains in such a complex environment is therefore highly possible, but due to the lack of biological material required for toxin analyses in the majority of samples, the presence of toxins was not assessed.

Despite the potential presence of cylindrospermopsin in the microbial mat from the cave entrance in Škocjanske jame (M-14, M-15), its presence could not be confirmed by downstream sequencing analysis (Table 1). The presence of cyanotoxins in caves should be further investigated, as cave microbial mat, i.e., lampenflora, has already demonstrated the presence of microcystins [28].

The *cyrJ* gene encodes a sulfotransferase, a tailoring reaction enzyme involved in the biosynthesis of cylindrospermopsin, which catalyses the sulphation of the C-12 atom [59]. This gene was proposed as a good candidate for a toxin assay because it is more unique than nonribosomal peptide synthetase (NRPS) and polyketide synthase (PKS) genes (such as some other genes in the cylindrospermopsin biosynthesis gene cluster, i.e., *cyrB*, *cyrC*, *cyrD*, *cyrE*, and *cyrF*) and is expected to have less cross-reactivity with other gene clusters containing these genes, commonly found in cyanobacteria (Mihali et al., 2008). The assay we used has already been successfully used for screening and sequencing of the *cyrJ* gene in isolated strains [60] and some environmental samples [61], but our results suggest that there might be some cross-reactivity with sulphur-oxidizing bacteria. However, this is not necessarily a consequence of low primer specificity but could also be due to a low annealing temperature used for PCR amplification.

This study could be expanded by a chemical analysis of cyanotoxins to confirm their presence. Metagenomic analysis by next-generation sequencing is another option for in-depth characterization of the community structure of cyanobacteria and evaluation of the cyanotoxin production potential, for example microcystin [62], saxitoxin [63], and cylindrospermopsin [64]. Our findings demonstrate the importance of selecting (q)PCR assays that are optimised for the analysis of environmental samples. Although the results of the study did not clearly confirm the presence of cyanotoxins in the studied habitats, this is an important consideration for public health in natural waters that may be used for drinking, recreational, or industrial purposes, such as public hot springs or tourist caves.

## 3. Conclusions

We performed molecular screening for the presence of cyanobacteria and cyanotoxin genes in samples from diverse habitats, covering a wide range of environmental conditions. Cyanobacteria were detected in various sample types: biofilms, microbial mats, and water. Molecular assays did not confirm the presence of genes for microcystin and saxitoxin synthesis in any of the samples. However, the analysis indicated the potential presence of cylindrospermopsin synthesis genes in a biofilm from a sulphidic spring. Further analysis is required to confirm the presence of cylindrospermopsins, as the cross-reactivity of the *cyrJ* assay with genomic DNA from the sulphur-oxidizing bacteria *Thiothrix* found in the sample makes the results inconclusive. Chemical analysis of cyanotoxins or metagenomic analysis could be performed to confirm the presence of cylindrospermopsins or to identify the organism carrying the target gene *cyrJ* in our samples. Several challenges were identified that need to be addressed in future research on cyanobacteria and their toxicity, in particular the lack of reference genes in databases and the specificity of molecular assays. Particular attention should be paid to the selection of molecular techniques and assays used to study mixed environmental communities, as cross-reactivity of molecular assays can bias results or on the other hand reveal new genomic traits in a complex sample. Ideally, a combination of different methods (e.g., molecular, chemical and morphological) targeting different properties of cyanobacteria and their metabolic characteristics should be used to gain a thorough and in-depth insight into environmental samples. Nevertheless, this study contributes to the limited data on the presence of cyanobacteria and cyanotoxin genes in underexplored habitats.

## 4. Material and Methods

### 4.1. Study Sites, Sampling and DNA Extraction

The samples were collected during several campaigns from randomly selected and accessible sites in different geographical locations from Croatia, Georgia, Italy, Serbia, and Slovenia in the period from 2011 to 2020 in the frame of occasional or regular monitoring (Table 1). Three different types of samples were collected: biofilm with nine samples (labelled B-01 to B-09), microbial mat with 19 samples (M-01 to M-19), and water with 33 samples (W-01 to W-33). We collected water and microbial mat samples from common, rare and extreme habitats that have not yet been analysed for cyanobacteria and cyanotoxins using a molecular approach and represent a temporal snapshot of the cyanobacterial community. Biofilm was considered to be a film of microorganisms embedded in extracellular polymers adhered on submerged surfaces or in aquatic environments. A microbial mat was considered to be a complex, multilayered structure of microorganisms collected from aerial and subaerial habitats.

Several samples (21%) originated from common habitats, i.e., aerial (M-01, M-02, M-03, M-04 and M-05) and aquatic habitats (W-03, W-05, W-08, W-09, W-11, W-17, W-18, W-22). Furthermore, 41% of the samples were collected from rare habitats, i.e., subaerial microbial mat from caves (M-06, M-07, M-08, M-09, M-10, M-11, M-12, M-13, M-14, M-15, M-16, M-17, M-18, M-19) and from waters in caves (W-07, W-23, W-24, W-25, W-26, W-27, W-28, W-29, W-30, W-31, W-33). The remaining 38% of the samples were from extreme habitats, i.e., alkaline environment (pH > 12; B-01 and W-15), saline spring (2 mg NaCl/L; W-01), low pH iron spring (pH ≤ 5.5; W-13 and W-14), sulphidic environment (B-02, B-03, B-04, B-05, B-06, B-07, B-08, B-09 and W-32), thermal spring (T ≥ 37 °C; W-02, W-04, W-06, W-10, W-12 and W-16), and samples from cave ice (W19, W20 and W21).

Initially, 11 additional water samples were included, but they were negative for both the 16S rRNA gene and phycocyanin intergenic spacer (PC-IGS) (q)PCR assays, and since the quality of the DNA could not be confirmed, they were excluded from further analyses. Biofilms were collected from alkaline and sulphidic springs and from a cave. Sulphidic springs emitted a characteristic odour of hydrogen sulphide. Multi-layered microbial mats in aerophytic habitats were sampled from buildings (façade, roof) and cave surfaces. In caves, this included lampenflora, i.e., the microbial mat around lamps from show caves [65], the microbial mat that developed in the cave entrance illuminated by sunlight, and a stromatolitic stalagmite. Stromatolitic stalagmites are calcareous deposits in cave entrances that are oriented towards the incoming sunlight due to the biolithogenic activity of cyanobacteria [66]. The water samples originated from different types of springs (acidic, alkaline, iron, saline, sulphidic, and thermal), borehole wells, cave seeping water, rivers, the sea, and melted ice from a cave. pH and temperature of the sampled waters were measured with a portable meter WTW Multiline 3420 (Xylem Analytics, Weilheim, Germany).

Samples were collected aseptically and transferred to a laboratory where the total community DNA was extracted. In the field, surfaces with biofilms and microbial mats were scraped with a sterile scalpel or knife and collected in sterile tubes. Water samples were collected in plastic bottles and filtered through 0.22 μm pore size filters (Merck Millipore, Burlington, MA, USA). Generally, 500 to 1000 mL of water volume was sufficient to obtain DNA, but some extractions required larger volumes, up to 10 L. Depending on the sample type and following the manufacturer’s instructions, three different kits were used for DNA isolation: PowerBiofilm DNA Isolation kit (MO BIO Laboratories, Carlsbad, CA, USA) for biofilm samples, PowerWater DNA Isolation kit (MO BIO Laboratories) for water samples, and NucleoSpin Soil kit (Macherey-Nagel, Düren, Germany) for microbial mats. DNA purity and concentration were determined spectrophotometrically using standard absorbance at 260 nm and 280 nm. After isolation DNA was stored at −20 °C until molecular analyses.

### 4.2. Molecular Analyses

The screening for cyanobacteria and cyanotoxins comprised several steps with different PCR and qPCR assays (Table 2). First, qPCR was performed with a cyanobacteria-specific 16S rRNA assay. Second, samples were screened with an end-point PCR assay targeting PC-IGS to confirm the results of the first step. Samples were further tested with five qPCR assays targeting genes involved in cyanotoxin synthesis: *cyrJ* (cylindrospermopsins), *sxtA* (saxitoxins), and *mcyE* (microcystins; *Dolichospermum*-, *Microcystis*-, and *Planktothrix*-specific). Finally, the PC-IGS and *cyrJ* regions were re-amplified by PCR in positive samples and sequenced.

#### 4.2.1. PCR Screening for PC-IGS

PCR amplification for PC-IGS (Table 2) was performed on the Mastercycler Nexus Gradient thermal cycler (Eppendorf, Hamburg, Germany). Reaction mixtures with a final volume of 10 µL were prepared in 0.2 mL 8-strip PCR tubes (Starlab, Hamburg, Germany), and contained 1× DreamTaq PCR buffer (Thermo Fisher Scientific, Waltham, MA, USA), 1 mM MgCl_2_, 0.3 µM each dNTP, 0.3 µM each forward and reverse primer, 0.25 U DreamTaq polymerase (Thermo Fisher Scientific), and 1 µL undiluted DNA template. PCR amplification included an initial denaturation of 3 min at 94 °C, followed by 35 cycles of 30 s at 94 °C, 30 s at 50 °C, and 1 min at 72 °C, with a final extension of 5 min at 72 °C. Positive controls (DNA from the cyanobacterial culture *Planktothrix agardhii* NIVA-CYA 126/8) and negative controls (nuclease-free water, Sigma-Aldrich, St. Louis, MO, USA) were included in each experiment. PCR products were visualised on a 0.8% agarose gel containing 0.5 × Tris-Borate-EDTA buffer, stained with Midori Green Advance DNA Stain (NIPPON Genetics EUROPE, Düren, Germany) at 120 V for 60 min. Then, 5 µL of the PCR product was loaded onto the gel together with *pstI*-digested λ DNA to determine the product size. In addition to the visual analysis of the gels, GelAnalyzer 19.1 [72] was used to determine band intensity and size.

#### 4.2.2. qPCR Reactions for Cyanobacteria and Toxin-Related Genes

The qPCR reactions were performed on the Applied Biosystems 7900HT qPCR cycler (Thermo Fisher Scientific) using SYBR Green I chemistry (Thermo Fisher Scientific). The reaction mixtures and amplification conditions were as described in Zupančič et al. (2021). Briefly, reactions were performed in triplicates with a final volume of 10 μL (5 μL of SYBR Green PCR Master Mix (Thermo Fisher Scientific), 0.3 μM (*Microcystis*-specific *mcyE* assay) or 0.9 μM (all other assays) of each primer, and 2 μL of 10-fold diluted DNA template. Primer sequences and references are listed in Table 2. Reactions were performed in clear 384-well PCR plates (Thermo Fisher Scientific) with MicroAmp™ optical adhesive film (Thermo Fisher Scientific). PCR amplification conditions included 2 min at 50 °C, an initial denaturation of 10 min at 95 °C, followed by 45 cycles of 15 s at 95 °C and 1 min at 60 °C, and a dissociation stage (15 s at 95 °C, 15 s at 60 °C, gradually increased to 95 °C). Positive controls (specific synthetic DNA fragments; [37]) and negative controls (nuclease-free water, Sigma-Aldrich) were included in each experiment.

Results were analysed with SDS software (version 2.4.1, Thermo Fisher Scientific) and data analyses were performed as described in Zupančič et al. (2021). Samples were considered positive if: (i) all three technical replicates were positive and (ii) the melting temperature (Tm) of the amplicon matched the Tm (±0.9 °C for 16S rRNA, ±0.5 °C for all other assays) of cyanobacterial cultures described in [37]. Samples with one or two positive replicates were considered potentially positive. Quantification of target cells (autotrophs based on 16S rRNA gene, and potential cylindrospermopsin producers based on cyrJ gene) was performed as described in [37]. For all positive and potentially positive samples, average Cq values were calculated from Cq values of all positive replicates. Relative quantification was performed using calibration curves approach. The calibration curves were generated from eight subsequent dilutions of DNA from cyanobacterial cultures as described in [37].

#### 4.2.3. Sequencing of PC-IGS and *cyrJ*

Amplification of PC-IGS and *cyrJ* genes (Table 2) for sequencing was performed on GeneAmp PCR system 9700 (Thermo Fisher Scientific). Reaction mixtures with a final volume of 30 µL contained 1x DreamTaq PCR buffer (Thermo Fisher Scientific), 1 mM MgCl_2_, 0.3 µM of each dNTP, 0.3 µM of each forward and reverse primer, 0.75 U Taq polymerase, and 3 µL undiluted DNA template. PCR amplification conditions included an initial denaturation of 3 min at 94 °C, followed by 35 cycles of 30 s at 94 °C, 30 s at 50 °C, and 1 min at 72 °C, with a final extension of 5 min at 72 °C. The PCR products were run on a 1.2% agarose gel with modified Tris-acetate-EDTA buffer (Montage DNA Gel Extraction Kit, Merck Millipore) stained with ethidium bromide at 100 V for 100 min. 25 µL of the product was loaded onto the gel together with GeneRuler 100 bp Plus DNA Ladder (Thermo Fisher Scientific) to determine the product size. Visible bands were extracted from the gel under UV light and purified using the Montage DNA Gel Extraction Kit (Merck Millipore). The products were sequenced using Sanger technology (Eurofins Genomics, Ebersberg, Germany). DNA sequences were analysed in BioEdit (v. 7.2.5), where forward and reverse reads were aligned (optimal global alignment), poor quality regions were removed, and trimmed sequences were aligned with BLAST [73] against the NCBI Nucleotide database [74]. The sequences were deposited in the NCBI GenBank database under the accession numbers PP061425–PP061428. DNA extracted from *Aphanizomenon ovalisporum* ILC-164 was used as a positive control for both PCR amplifications and sequenced together with the samples, and high quality reads were obtained from both target regions. For PC-IGS, the 573 bp long read showed 100% identity with PC-IGS genes from other *A. ovalisporum* sequences deposited in NCBI Nucleotide (strains UAM287, UAM289, APH033B, KA1.1, UAM291, and UAM290, as well as *Anabaena bergii* ANA366B). For *cyrJ*, the 488 bp long read showed 100% identity with *cyrJ* genes from strains ILC-164 and LK_11a.

### 4.3. Microscopy of Sulphidic Biofilm

Although the focus of this work was on molecular methods used for screening, microscopy was employed as an additional tool to observe morphological characteristics of one sulphidic biofilm sample. The biofilm from Žveplenica sulphidic spring was sampled after molecular analysis that indicated the presence of potential cylindrospermopsin producers in the sample. Microscopic observation of the fresh material was performed under phase contrast with 400×, 600× and 1000× magnification (Nikon Eclipse TE300, Tokyo, Japan). The autotrophic community was determined according to the taxonomic key in [75,76,77].

## Figures and Tables

**Table 1:** List of samples collected in different locations (country, location and coordinates are given) on different sampling dates. Biofilm, microbial mat and water samples were collected in diverse environments, some of them extreme (high/low temperature and/or pH). All samples were tested with PC-IGS PCR assay, and 16S rRNA gene, *cyrJ*, *mcyE*, and *sxtA* qPCR assays, and results are reported as + (positive) and − (negative). Quantitative results are available in Supplementary Tables S1 and S2. PC-IGS and *cyrJ* amplicons of selected samples were sequenced, and the most similar genus is indicated.

Sample	Country	Location	Coordinates	Sampling Date [dd/mm/yyyy]	Sample Type	Description	T [°C]	pH	qPCR (16S rRNA)	PCR PC-IGS	Seq. PC-IGS (Genus)	qPCR *cyrJ*	Seq. *cyrJ*	qPCR *stxA*	qPCR *mcyE* (*Dol.*)	qPCR *mcyE* (*Mic.*)	qPCR *mcyE* (*Pla.*)
B-01	Serbia	Bela voda, Zlatibor	43°42′13″ N, 19°34′43″ E	29/09/2011	biofilm	alkaline spring	15.6	12.40	+	+	(+)	−	NS	−	−	−	−
B-02	Slovenia	Hajnsko	45°12′22″ N, 15°35′26″ E	13/04/2016	biofilm	sulphidic spring	13.6	7.13	+	−	NS	−	NS	−	−	−	−
B-03	Slovenia	Matijeva jama	45°41′21″ N, 14°15′53″ E	31/08/2016	biofilm	biofilm in a cave	NA	NA	+	−	NS	−	NS	−	−	−	−
B-04	Slovenia	Smrdljivec	45°39′48″ N, 13°59′57″ E	24/08/2017	biofilm	sulphidic spring	19.7	7.28	+	−	NS	−	NS	−	−	−	−
B-05	Slovenia	Smrdljivec	45°39′48″ N, 13°59′57″ E	24/08/2017	biofilm	sulphidic spring	19.7	7.28	+	−	NS	−	NS	−	−	−	−
B-06	Slovenia	Smrdljivec	45°39′48″ N, 13°59′57″ E	24/08/2017	biofilm	sulphidic spring	19.7	7.28	+	−	NS	−	NS	−	−	−	−
B-07	Slovenia	Riharjev studenec	46°18′50″ N, 14°42′07″ E	22/03/2016	biofilm	sulphidic spring	10.2	7.76	+	−	NS	(+)	+ *Thio-thrix*	−	−	−	−
B-08 *	Slovenia	Žveplenica	46°03′59″ N, 13°49′38″ E	10/04/2016	biofilm	sulphidic spring	10.5	7.54	+	−	NS	+	(+)	−	−	−	−
B-09	Slovenia	Žvepovnik	46°21′00″ N, 14°50′43″ E	22/03/2016	biofilm	sulphidic spring	10.4	7.32	+	−	NS	−	NS	−	−	−	−
M-01	Slovenia	Ljubljana	46°02′59″ N, 14°29′58″ E	08/06/2015	microbial mat	facade, city centre	NA	NA	+	−	NS	−	NS	−	−	−	−
M-02	Slovenia	Ljubljana	46°04′57″ N, 14°30′47″ E	08/06/2015	microbial mat	facade, city outskirt	NA	NA	+	−	NS	−	NS	−	−	−	−
M-03	Slovenia	Ljubljana	46°04′57″ N, 14°30′47″ E	08/06/2015	microbial mat	facade, city outskirt	NA	NA	+	−	NS	−	NS	−	−	−	−
M-04	Slovenia	Ljubljana	46°04′14″ N, 14°30′51″ E	05/06/2015	microbial mat	roof, city outskirt	NA	NA	+	−	NS	−	NS	−	−	−	−
M-05	Slovenia	Ljubljana	46°04′14″ N, 14°30′51″ E	05/06/2015	microbial mat	roof, city outskirt	NA	NA	+	−	NS	−	NS	−	−	−	−
M-06	Slovenia	Matijeva jama	45°41′21″ N, 14°15′53″ E	08/07/2019	microbial mat	microbial mat at a cave entrance	NA	NA	+	+	−	−	NS	−	−	−	−
M-07	Slovenia	Postojnska jama	45°46′59″ N, 14°12′14″ E	24/01/2020	microbial mat	lampenflora in a cave	NA	NA	+	−	NS	−	NS	−	−	−	−
M-08	Slovenia	Postojnska jama	45°46′59″ N, 14°12′14″ E	24/01/2020	microbial mat	lampenflora in a cave	NA	NA	+	−	NS	−	NS	−	−	−	−
M-09	Slovenia	Postojnska jama	45°46′59″ N, 14°12′14″ E	24/01/2020	microbial mat	lampenflora in a cave	NA	NA	+	−	NS	−	NS	−	−	−	−
M-10	Slovenia	Postojnska jama	45°46′59″ N, 14°12′14″ E	24/01/2020	microbial mat	lampenflora in a cave	NA	NA	+	+	(+)	−	NS	−	−	−	−
M-11	Slovenia	Postojnska jama	45°46′59″ N, 14°12′14″ E	24/01/2020	microbial mat	lampenflora in a cave	NA	NA	+	+	−	−	NS	−	−	−	−
M-12 *	Slovenia	Škocjanske jame	45°39′57″ N, 13°59′24″ E	08/04/2016	microbial mat	microbial mat at a cave entrance	NA	NA	+	−	NS	−	NS	−	−	−	−
M-13	Slovenia	Škocjanske jame	45°39′57″ N, 13°59′24″ E	23/01/2020	microbial mat	microbial mat at a cave entrance	NA	NA	+	+	−	−	NS	−	−	−	−
M-14	Slovenia	Škocjanske jame	45°39′57″ N, 13°59′24″ E	23/01/2020	microbial mat	microbial mat at a cave entrance	NA	NA	+	+	(+)	(+)	−	−	−	−	−
M-15	Slovenia	Škocjanske jame	45°39′57″ N, 13°59′24″ E	23/01/2020	microbial mat	microbial mat at a cave entrance	NA	NA	+	+	(+)	(+)	−	−	−	−	−
M-16	Slovenia	Škocjanske jame	45°39′57″ N, 13°59′24″ E	23/01/2020	microbial mat	microbial mat at a cave entrance	NA	NA	+	+	−	−	NS	−	−	−	−
M-17	Slovenia	Škocjanske jame	45°39′57″ N, 13°59′24″ E	23/01/2020	microbial mat	microbial mat at a cave entrance	NA	NA	+	+	+ *Cyano-thece*	−	NS	−	−	−	−
M-18 *	Slovenia	Škocjanske jame	45°39′57″ N, 13°59′24″ E	08/04/2016	microbial mat	microbial mat at a cave entrance	NA	NA	+	+	−	−	NS	−	−	−	−
M-19 *	Slovenia	Škocjanske jame	45°39′57″ N, 13°59′24″ E	08/04/2016	microbial mat	stromatolitic stalagmite at a cave entrance	NA	NA	+	+	+ *Calothrix*	−	NS	−	−	−	−
W-01	Croatia	Slanci, Slanje	46°13′48″ N, 16°32′56″ E	24/03/2019	water	saline spring #	17.6	7.97	+	+	+ *Synecho-cystis*	−	NS	−	−	−	−
W-02	Georgia	Analisopeli	41°49′15″ N, 41°47′54″ E	13/07/2012	water	discharge from a borehole well	36.9	7.50	+	−	NS	−	NS	−	−	−	−
W-03	Georgia	Sighnaghi	41°36′23″ N, 45°56′02″ E	30/06/2012	water	spring	12.3	7.50	+	−	NS	−	NS	−	−	−	−
W-04	Georgia	Tbilisi, Lisi	41°43′13″ N, 44°45′30″ E	02/07/2012	water	discharge from a borehole well	63.4	7.50	+	−	NS	−	NS	−	−	−	−
W-05	Georgia	Tbilisi, Ortachala	41°40′25″ N, 44°50′30″ E	04/07/2012	water	discharge from a borehole well	21.5	8.00	+	−	NS	−	NS	−	−	−	−
W-06	Georgia	Vardzia Cave Monastery	41°23′30″ N, 43°18′33″ E	08/07/2012	water	discharge from a borehole well	51.0	9.30	(+)	−	NS	−	NS	−	−	−	−
W-07	Georgia	Vardzia Cave Monastery	41°22′52″ N, 43°17′03″ E	08/07/2012	water	seeping water in a cave	10.5	7.00	(+)	−	NS	−	NS	−	−	−	−
W-08	Italy	Soča/Isonzo River	45°56′17″ N, 13°36′05″ E	20/06/2019	water	river	16.4	8.30	+	−	NS	−	NS	−	−	−	−
W-09	Italy	Vipava/Vipacco River	45°53′17″ N, 13°35′27″ E	20/06/2019	water	river	20.9	8.30	+	−	NS	−	NS	−	−	−	−
W-10	Italy	Tržič/Monfalcone	45°47′32″ N, 13°33′57″ E	20/06/2019	water	thermal spring	37.4	7.10	+	−	NS	−	NS	−	−	−	−
W-11	Italy	Adriatic sea	45°46′51″ N, 13°32′23″ E	20/06/2019	water	sea	25.8	8.20	+	−	NS	−	NS	−	−	−	−
W-12	Italy	Tržič/Monfalcone	45°47′31″ N, 13°33′56″ E	20/06/2019	water	discharge from a borehole well	39.2	7.00	(+)	−	NS	−	NS	−	−	−	−
W-13	Serbia	Crveno vrelo, Đavolja Varoš	42°59′26″ N, 21°23′48″ E	28/09/2012	water	iron spring	16.9	5.50	+	−	NS	−	NS	−	−	−	−
W-14	Serbia	Đavolje vrelo, Đavolja Varoš	42°59′19″ N, 21°23′35″ E	28/09/2012	water	iron spring	25.5	2.50	+	−	NS	−	NS	−	−	−	−
W-15	Serbia	Mokra Gora, Zlatibor	43°47′28″ N, 19°32′13″ E	28/09/2010	water	alkaline spring	15.4	12.03	(+)	−	NS	−	NS	−	−	−	−
W-16	Serbia	Vranjska Banja	42°33′00″ N, 22°00′23″ E	29/09/2012	water	thermal spring	83.0	8.50	(+)	−	NS	−	NS	−	−	−	−
W-17	Slovenia	Klariči	45°48′49″ N, 13°35′54″ E	20/06/2019	water	discharge from a borehole well	14.3	7.50	+	−	NS	−	NS	−	−	−	−
W-18	Slovenia	Reka River	45°39′21″ N, 14°03′21″ E	20/06/2019	water	river	20.2	8.50	+	−	NS	−	NS	−	−	−	(+)
W-19	Slovenia	Paradana	45°59′19″ N, 13°50′41″ E	09/05/2016 04/06/2016 **	water	ice from cave	NA	8.21 **	+	NA	NS	−	NS	−	−	−	−
W-20	Slovenia	Paradana	45°59′19″ N, 13°50′41″ E	09/05/2016 04/06/2016 **	water	ice from cave	NA	8.62 **	+	−	NS	−	NS	−	−	−	−
W-21	Slovenia	Paradana	45°59′19″ N, 13°50′41″ E	09/05/2016 04/06/2016 **	water	ice from cave	NA	8.46 **	+	−	NS	−	NS	−	−	−	−
W-22	Slovenia	Pivka River, Postojna	45°46′55″ N, 14°12′14″ E	02/07/2019	water	river before ponor in a cave	22.6	7.59	+	−	NS	−	NS	−	−	−	−
W-23	Slovenia	Planinska jama	45°49′15″ N, 14°14′48″ E	17/07/2017 15/11/2023 **	water	seeping water in a cave	11.2 **	8.12 **	+	−	NS	−	NS	−	−	−	−
W-24	Slovenia	Planinska jama	45°49′15″ N, 14°14′48″ E	11/10/2017 15/11/2023 **	water	seeping water in a cave	11.2 **	8.12 **	+	−	NS	−	NS	−	−	−	−
W-25	Slovenia	Planinska jama	45°49′15″ N, 14°14′48″ E	11/10/2017 15/11/2023 **	water	seeping water in a cave	10.9 **	7.95 **	+	−	NS	−	NS	−	−	−	−
W-26	Slovenia	Planinska jama	45°49′15″ N, 14°14′48″ E	11/10/2017 15/11/2023 **	water	seeping water in a cave	10.9 **	7.88 **	+	−	NS	−	NS	−	−	−	−
W-27	Slovenia	Planinska jama	45°49′15″ N, 14°14′48″ E	14/11/2017 15/11/2023 **	water	seeping water in a cave	11.2 **	8.12 **	+	−	NS	−	NS	−	−	−	−
W-28	Slovenia	Planinska jama	45°49′15″ N, 14°14′48″ E	14/11/2017 15/11/2023 **	water	seeping water in a cave	10.9 **	7.95 **	+	−	NS	−	NS	−	−	−	−
W-29	Slovenia	Planinska jama	45°49′15″ N, 14°14′48″ E	14/11/2017 15/11/2023 **	water	seeping water in a cave	10.9 **	7.88 **	+	−	NS	−	NS	−	−	−	−
W-30	Slovenia	Postojnska jama	45°46′59″ N, 14°12′14″ E	11/10/2017	water	seeping water in a cave	10.6	8.04	+	−	NS	−	NS	−	−	−	−
W-31	Slovenia	Postojnska jama	45°46′59″ N, 14°12′14″ E	14/11/2017	water	seeping water in a cave	10.6	8.04	+	−	NS	−	NS	−	−	−	−
W-32	Slovenia	Smrdljivec	45°39′48″ N, 13°59′57″ E	18/07/2017	water	sulphidic spring	21.5	7.28	+	−	NS	−	NS	−	−	−	−
W-33	Slovenia	Škocjanske jame	45°39′57″ N, 13°59′24″ E	13/12/2017	water	seeping water in a cave	18.7	8.16	+	−	NS	−	NS	−	−	−	−

PC-IGS—phycocyanin intergenic spacer; 16S—16S RNA gene (cyanobacteria-specific); qPCR results: + 3/3 technical replicates positive; (+) 1 or 2 replicates positive; Seq (sequencing): + target gene confirmed and taxa determined; (+) target gene confirmed, but taxa could not be determined; Dol.—*Dolichospermum*; Mic.—*Microcystis*; Pla.—*Planktothrix*; NA—not analysed; NS—not sequenced; * two technical replicates (sample aliquots) were analysed and the results were merged; ** date of measurements differed from sampling; # 2 mg/L (unpublished).

**Table 2:** qPCR and PCR assays used in this study.

Type of Analysis	Target Organisms	Target Gene	Primer Label	Nucleotide Sequence (5′ → 3′)	Amplicon Length [bp]	Reference
qPCR	Cyanobacteria	16S rRNA	cyano-real16S-F	AGC CAC ACT GGG ACT GAG ACA	73	[67]
			cyano-real16S-R	TCG CCC ATT GCG GAA A		
PCR, sequencing	Cyanobacteria	PC-IGS	PCβF	GGC TGC TTG TTT ACG CGA CA	500–740	[35]
			PCαR	CCA GTA CCA CCA GCA ACT AA		
qPCR	Cylindrospermopsin-producers	*cyrJ*	cyrJ207-F	CCC CTA CAA CCT GAC AAA GCT T	77	[68]
			cyrJ207-R	CCC GCC TGT CAT AGA TGC A		
PCR, sequencing	Cylindrospermopsin-producers	*cyrJ*	cynsulF	ACT TCT CTC CTT TCC CTA TC	586	[59]
			cylnamR	GAG TGA AAA TGC GTA GAA CTT G		
qPCR	Saxitoxin-producers	*sxtA*	sxtA-F	GAT GAC GGA GTA TTT GAA GC	125	[67]
			sxtA-R	CTG CAT CTT CTG GAC GGT AA		
qPCR	Microcystin- producers (genus *Dolichospermum*)	*mcyE*	mcyE-F2	GAA ATT TGT GTA GAA GGT GC	247	[69]
			AnamcyE-12R	CAA TCT CGG TAT AGC GGC		[70]
qPCR	Microcystin- producers (genus *Microcystis*)	*mcyE*	mcyE-F2	GAA ATT TGT GTA GAA GGT GC	247	[69]
			MicmcyE-R8	CAA TGG GAG CAT AAC GAG		[70]
qPCR	Microcystin- producers (genus *Planktothrix*)	*mcyE*	mcyE-F2	GAA ATT TGT GTA GAA GGT GC	249	[69]
			PlamcyE-R3	CTC AAT CTG AGG ATA ACG AT		[71]

## Data Availability

Data are available from the corresponding authors upon request. The nucleotide sequences produced in this study were deposited in the NCBI GenBank database under the accession numbers PP061425–PP061428.

## Supplementary Materials

The following supporting information can be downloaded at: https://www.mdpi.com/article/10.3390/toxins16080333/s1, Table S1: qPCR results and the corresponding calculated target cell concentrations obtained with the cyanobacteria-specific 16S rRNA assay targeting autotrophs; Table S2: qPCR results and the corresponding calculated target cell concentrations obtained with the *cyrJ* assay targeting potential cylindrospermopsin producers.
