# Supplementary material for: Molecular Screening for Cyanobacteria and Their Cyanotoxin Potential in Diverse Habitats
Source: Toxins (Basel). 2024 Jul 27;16(8):333. doi: 10.3390/toxins16080333 (PMC11360522; doi:10.3390/toxins16080333)
Supplement: Supplementary file 1 [file toxins-16-00333-s001.zip › toxins-3058265-supplementary.pdf]

Article

Molecular Screening for Cyanobacteria and Their Cyanotoxin Potential in Diverse Habitats

Maša Jablonska [1,2,*], Tina Eleršek [1], Polona Kogovšek [3], Sara Skok [4], Andreea Oarga-Mulec [5] and Janez Mulec [4,6,*]

1 Department of Genetic Toxicology and Cancer Biology, National Institute of Biology, 1000 Ljubljana, Slovenia; tina.elersek@nib.si
2 Biotechnical Faculty, University of Ljubljana, 1000 Ljubljana, Slovenia
3 Department of Biotechnology and Systems Biology, National Institute of Biology, 1000 Ljubljana, Slovenia; polona.kogovsek@nib.si
4 Karst Research Institute, Research Centre of the Slovenian Academy of Sciences and Arts, 6230 Postojna, Slovenia; sara.skok@zrc-sazu.si
5 Materials Research Laboratory, University of Nova Gorica, 5000 Nova Gorica, Slovenia; andreea.oarga@ung.si
6 UNESCO Chair on Karst Education, University of Nova Gorica, 5271 Vipava, Slovenia
* Correspondence: masa.jablonska@nib.si (M.J.), janez.mulec@zrc-sazu.si (J.M.)

SUPPLEMENTARY MATERIAL

Supplementary Table S1. qPCR results and the corresponding calculated target cell concentrations obtained with the cyanobacteria-specific 16S rRNA assay targeting autotrophs. For each sample, three replicates were tested, and the average quantification cycle (Cq) values and average melting temperatures (Tm) are given. < LOQ – positive but below limit of quantification; * two technical replicates (sample aliquots) were analysed and the results were averaged; ** only one or two out of three replicates (DNA aliquots) tested were positive.

Sample	Average Cq	Average Tm [°C]	Target cell concentration [cells/µL DNA]
B-01	30.91	81.17	< LOQ
B-02	20.62	80.93	5.30×10^4
B-03	27.42	80.63	1.03×10^3
B-04	30.03	81.37	2.26×10^2
B-05	28.23	80.90	6.42×10^2
B-06	32.82	80.80	< LOQ
B-07	22.84	81.30	1.47×10^4
B-08*	25.15	81.00	4.35×10^3
B-09	22.84	81.30	1.47×10^4
M-01	21.01	80.93	4.24×10^4
M-02	29.06	81.00	3.96×10^2
M-03	21.02	80.93	4.20×10^4
M-04	18.61	81.10	1.70×10^5
M-05	23.50	81.20	1.00×10^4
M-06	20.97	81.70	4.34×10^4
M-07	17.83	81.40	2.68×10^5
M-08	17.80	81.50	2.73×10^5
M-09	17.24	81.57	3.77×10^5
M-10	19.15	81.40	1.25×10^5
M-11	16.31	81.63	6.46×10^5
M-12*	24.68	81.50	6.41×10^3
M-13	16.28	81.50	6.58×10^5
M-14	16.73	81.37	5.07×10^5
M-15	17.97	81.50	2.47×10^5
M-16	17.30	81.50	3.64×10^5
M-17	16.32	81.50	6.44×10^5
M-18*	21.39	81.50	4.43×10^4
M-19*	19.70	81.42	1.08×10^5
W-01	21.12	81.80	3.97×10^4
W-02	34.35	81.40	< LOQ
W-03	34.93	81.37	< LOQ
W-04	34.48	81.07	< LOQ
W-05	32.98	80.70	< LOQ
W-06**	34.67	81.20	1.53×10^1
W-07**	34.53	80.85	1.66×10^1
W-08	26.82	80.80	1.45×10^3

Supplementary Table S1. (continued)

Sample	Average Cq	Average Tm [°C]	Target cell concentration [cells/μL DNA]
W-09	18.45	81.00	1.87×10^5
W-10	25.82	80.90	2.59×10^3
W-11	19.07	81.57	1.31×10^5
W-12*	34.29	80.70	1.91×10^1
W-13	34.96	80.97	< LOQ
W-14	27.55	80.97	9.52×10^2
W-15**	34.35	81.00	1.84×10^1
W-16**	34.16	81.20	2.05×10^1
W-17	24.72	81.20	4.92×10^3
W-18	20.98	81.20	4.31×10^4
W-19	30.82	81.17	< LOQ
W-20	31.80	81.17	< LOQ
W-21	28.73	81.00	4.80×10^2
W-22	19.95	81.20	7.83×10^4
W-23	27.66	81.17	8.96×10^2
W-24	26.50	80.87	1.75×10^3
W-25	25.75	80.80	2.71×10^3
W-26	26.61	80.80	1.64×10^3
W-27	26.83	80.90	1.45×10^3
W-28	25.99	80.90	2.35×10^3
W-29	25.37	80.80	3.38×10^3
W-30	30.77	81.00	< LOQ
W-31	33.81	81.07	< LOQ
W-32	25.17	81.00	3.79×10^3
W-33	27.94	81.60	7.62×10^2

Supplementary Table S2. qPCR results and the corresponding calculated target cell concentrations obtained with the *cyrJ* assay targeting potential cylindrospermopsin producers. For each sample, three replicates were tested, and the average quantification cycle (Cq) values and average melting temperatures (Tm) are given. * two technical replicates (sample aliquots) were analysed and the results were averaged; ** only one or two out of three replicates (DNA aliquots) tested were positive.

**Sample**	**Average Cq**	**Average Tm** [°C]	**Target cell concentration [cells/µL DNA]**
B-07*	31.65	79.60	2.67×10^{2}
B-08**	29.69	79.95	8.01×10^{2}
M-14*	33.32	79.80	1.08×10^{2}
M-15*	34.18	80.10	6.71×10^{1}
